# Perspectives on smokefree prison policy among people in custody in Scotland

**DOI:** 10.1108/IJPH-12-2019-0065

**Published:** 2020-07-29

**Authors:** Ashley Brown, Douglas Eadie, Richard Purves, Andrea Mohan, Kate Hunt

**Affiliations:** 1 Institute for Social Marketing and Health, University of Stirling, Stirling, UK; 2 School of Nursing and Health Sciences, University of Dundee, Dundee, UK; 3 Institute for Social Marketing and Health, University of Stirling, Stirling, UK

**Keywords:** Health in prison, Offender health, Prisoners, Public health, Prison, Health policy

## Abstract

**Purpose:**

This paper aims to explore smokefree prison policy, from the perspective of people in custody in Scotland.

**Design/methodology/approach:**

In total, 77 people in custody in Scotland were interviewed in the period leading up to implementation of a nationwide prison smokefree policy. Data were thematically analysed to identify the diversity of views and experiences.

**Findings:**

Participants described a widespread awareness in prisons of plans to implement a smokefree policy from 30 November 2018. Opinions about smokefree prisons varied among participants based on perceptions of the fairness, and anticipated positive and negative consequences of removing tobacco from prisons. At the time of the interviews, people in custody were responding to the impending smokefree policy, either by proactively preparing for the smokefree rule change or by deploying avoidance strategies. Participants described opportunities and challenges for implementing smokefree policy in prisons across three main themes: the role of smoking in prison, prison smoking cessation services and motivations for quitting smoking among people in custody.

**Originality/value:**

This study exploring smokefree prisons from the perspectives of people in custody has several novel features which extend the evidence base. The findings highlight measures for jurisdictions to consider when planning to prohibit smoking in their prisons in the future. These include the need for evidence-based smoking cessation support in advance of smokefree policy, effective communication campaigns, consideration of broader structural determinants of health in prison and ongoing measures to reduce rates of return to smoking post release.

## Introduction

Smoking is a leading cause of preventable illness and death globally (Reitsma *et al.*, 2017). Reducing uptake and use of tobacco and exposures to second-hand smoke (SHS) is a major health priority, resulting in the implementation of tobacco control measures in countries around the world (World Health Organisation, 2008). While there have been significant reductions in smoking at a population level, people in custody (prisoners who have been convicted or on remand awaiting trial) are one group among whom tobacco use has been very high (Spaulding *et al.*, 2018). Yet, there is evidence that not only do many people in custody take up (again) or increase smoking while in prison (Baybutt *et al.*, 2014) but also most people in custody who smoke express an interest in trying to quit (Ahalt *et al.*, 2019; Scottish Prison Service, 2015; Valera *et al.*, 2019).

Interventions providing (free) behavioural support and/or nicotine replacement therapy (NRT) to people in custody who smoke is one potential way in which countries may support smoking cessation in prison. A review evaluating the effectiveness of smoking cessation interventions in prisons, which included ten quantitative studies of varying methodological quality, found evidence that smoking cessation interventions “can significantly increase the likelihood of quitting in prison and increase abstinence post release” (de Andrade and Kinner, 2016, p. 1). However, challenges in respect of scaling up interventions or encouraging uptake among people in custody may limit the reach of prison smoking cessation interventions, potentially because of strong social norms (tobacco use is normative) and the linking of smoking with enjoyment and stress relief in a setting in which people may believe there are few alternatives. Another way in which jurisdictions may try to reduce smoking and SHS exposures in prison is via implementation of partial or total smokefree policy. The same review by de Andrade and Kinner (2016) found that partial smokefree policy can lead to significant reductions in the number of cigarettes smoked per day, while total smokefree policy can increase rates of smoking abstinence. However, the findings suggest non-compliance with smokefree rules and relapse to smoking following liberation may potentially constrain longer-term health benefits (de Andrade and Kinner, 2016). For this reason, several commenters have advocated for smokefree prison policy to be accompanied by evidence-based cessation interventions which seek to strengthen smokers’ readiness to change and support individuals to sustain abstinence in prison and beyond (Butler *et al.*, 2007; Puljevic and Segan, 2018; Ritter, 2014).

A review of 12 studies (Djachenko *et al.*, 2015, p. 43) exploring smoking cessation among men in custody identified that factors such as “a ‘pro-smoking’ culture in prison and the entrenched role of tobacco in prison society”, places constraints on prison and health-care resources, and lack of prioritisation of smoking over other health issues, could work against measures to promote smoking behaviour change in the prison setting. Several of these studies also identified opportunities for increasing quitting in prison by connecting with some people’s interests in making positive lifestyle changes during prison sentences.

Within the UK, prisons in Wales, England and Scotland have become smokefree in recent years (Selous, 2015; Scottish Prison Service, 2017a). The intention to implement a total smokefree policy in all Scottish prisons from 30 November 2018 was announced in July 2017 (Scottish Prison Service, 2017a), in part in response to evidence on levels of SHS in prisons (Semple *et al.*, 2017). In the UK, smokefree prison policies were implemented in a unique context; free smoking cessation treatment and behavioural support were available in prisons prior to smoking becoming prohibited. A national prison smoking cessation service specification for Scotland was published in 2015 that recommended that services in prisons, like those in the wider community, should provide “*a combination of [free] multisession intensive behavioural support together with pharmacology*” (NHS Health Scotland, 2015, p. 8), including use of carbon monoxide (CO) monitoring to validate progress and encourage quitting. A detailed description of service delivery recommendations can be found elsewhere (NHS Health Scotland, 2015). In the period between the announcement in July 2017 and the legislation for smokefree prisons coming into force (30 November 2018), prison and health services worked in collaboration to maximise existing service performance, and develop a new “smokefree prisons pathway” (NHS Health Scotland, 2018) to support people in custody to manage without tobacco following the change of prison smoking rules.

It is important to understand smokefree prison policy from the perspectives of people in custody, to support successful implementation and ongoing management of such policies in jurisdictions who may remove tobacco from their prisons in the future. Two qualitative studies have reported the views of people in custody in England (Woodall and Tattersfield, 2017; Dugdale *et al.*, 2019). This paper is based on qualitative data collected from people in custody in Scotland between November 2017 and June 2018, when the sale and use of tobacco was still permitted in prison, and aims to extend previous studies. The research is unique in being one part of a comprehensive multi-methods, three-phase country-wide evaluation (the Tobacco In Prisons study) of the development, implementation and outcomes of smokefree prisons across a national prison system. Also of note is that Scotland differed from other prison systems (e.g. England and Wales) by introducing smokefree legislation in all of its prisons on the same date; interviews were conducted during the anticipatory period after it had been announced that all prisons would become smokefree; the interviews included a sub-sample of participants who were using cessation services.

## Methods

The Tobacco in Prisons study, a three-phase multimethod study, has been evaluating the process and outcomes of the introduction of a comprehensive smokefree policy in Scottish prisons. Phase 1 sought to understand the situation across the Scottish prison system before any decision had been made on whether or when to change existing regulations about smoking (September 2016-July 2017). Phase 2 began after the announcement in mid-July 2017 that Scotland’s prisons would all go smokefree in November 2018. Phase 3 began in December 2018 and is assessing outcomes of the policy. Results from Phase 1 qualitative and survey work on staff and prisoner views have been published (Brown *et al.*, 2018), as have reports of air quality across all prisons in Phase 1 (Semple *et al.*, 2017) which informed decisions about smokefree policy, and in the week spanning the implementation of the smokefree policy on 30 November 2018 (Semple *et al.*, 2019).

Two of the study’s work packages (WPs) have used qualitative interviews to explore issues surrounding smoking, smoking cessation and smoking restrictions in prisons from the perspective of a key group, namely, people in custody. This paper presents an analysis of these two complementary qualitative datasets (referred to as WP4 and WP5), both collected in Phase 2, i.e. the period between announcement and the implementation of the prison smokefree policy in Scotland, and before the introduction of rechargeable e-cigarettes in prisons. WP4 interviews took place from November 2017–January 2018, while the WP5 interviews were conducted from May to June 2018. At the time of both sets of interviews, people in custody were permitted to purchase tobacco from the prison shop (“canteen”) and to smoke tobacco in their rooms (cells) and during outdoor recreation. The decision to remove tobacco from prisons from 30th November 2018 had thus been announced 5–12 months prior to the interviews which form the basis of this paper.

This study was approved by the Scottish Prison Service Research Access and Ethics Committee and University of Glasgow’s College of Social Science Ethics Committee (references for interviews with people in custody 400150214 and 400160041).

### Sample and recruitment

For both WPs, we conducted these Phase 2 interviews with people in custody in five prisons in Scotland, selected in discussion with the SPS to represent a range of prisoner groups and prison environments. Interviews in a sixth prison were also conducted as part of WP4. People in custody were recruited via a point of contact within each prison. These contacts were asked to provide information about the study to a sample of people in custody who held particular characteristics. For WP4, the researchers sought to interview a mixture of men/women and people on shorter and longer sentences. For WP5, the researchers aimed to interview people in custody who had experience of using smoking cessation services while in prison. In total, 77 participants took part in the interviews included in this analysis, 33 interviewed for WP4 and 44 for WP5. Most were current smokers or current users of the prison smoking cessation service. A minority were ex-smokers, most of whom reported stopping smoking within the last year. Self-reported use of prison smoking cessation services was ∼75% for WP5 participants; the equivalent figure for WP4 participants was 45%. As shown in Table 1, the achieved samples for both WPs were diverse with respect to: sex, remanded/convicted status and sentence length. Information on other socio-demographic characteristics were not collected.

### Data collection

In total, 75 people in custody were interviewed one-to-one; two participants opted to take part in a paired interview. Researchers conducted the interviews in rooms chosen by the point of contact to give people in custody the opportunity to express their views in private. Topic guides covered similar but not identical general topics for WPs 4 and 5. The content of each guide was shaped by the study objectives and literature and was refined over multiple iterations based on feedback from the research team. Both covered: background and time in prison; smoking history; experiences of smoking tobacco and changes over time (particularly in light of the impending smokefree policy); the prison context and smoking; restrictions on smoking in prisons and opinions of these; views on what may help or hinder successful implementation of the prison smokefree policy; opinions and experiences of quitting smoking in the prison setting; and views on e-cigarettes in prisons. The time dedicated to topics differed between the two sets of interviews: the WP4 interviews sought to explore a wide range of issues in relation to smoking, smoking cessation and smoking restrictions in the prison setting, while the WP5 interviews in particular sought participants’ opinions and experiences of prison smoking cessation services and other support for quitting within prison. Researchers conducting the interviews formulated questions using their own words, probed for further detail where necessary and asked participants to raise any issues which they considered relevant.

### Data management and analysis

All interviews were audio-recorded with the participants’ permission and transcribed by a professional transcription company. Data were thematically analysed, broadly adhering to the principles described by Spencer *et al.* (2014) which share similarities with the approach to thematic analysis developed by Braun and Clarke (2006). Emerging themes from the WP4 and WP5 interviews with people in custody were independently identified (using different approaches) by AB and DE/RP/AM, respectively. For WP4, data summaries for every transcript were entered by AB into the cells of a framework grid (rows=interviews, columns= themes) in Nvivo prior to detailed analysis. The equivalent step for WP5 involved indexing (but not summarising) transcripts against a descriptive coding scheme using Nvivo. The thematic framework for WP4 and the descriptive coding scheme for WP5 were developed using a combination of inductive (e.g. detailed reading of transcripts) and deductive techniques (e.g. reviewing of research questions, topic guide and literature). The authors used the framework grid or coded data and whole transcripts to identify emerging themes from the two data sets, paying attention to the range and diversity of responses. Over multiple iterations data were grouped into categories according to their perceived similarities or differences to create themes and sub-themes. The emergent themes identified in the two data sets were substantively very similar: the research team therefore decided to combine data sets for final analysis and reporting. AB led on creating final themes and sub-themes by combining/separating and refining initial themes identified during the previous stage of analysis. Final interpretations were carefully checked against data from both WPs, and discussed and agreed on by all authors. Quotes are included to illustrate key findings, indicating prison code, participant ID and participant smoking status [smoker/ex-smoker/(current) service user of the prison smoking cessation service].

## Results

As illustrated in the quote below, interviews were conducted at a time where there was reportedly widespread awareness among people in custody (and staff) that a comprehensive smokefree policy would be implemented in Scottish prisons in 6–12 months’ time (i.e. on 30 November 2018):

WP5.A08.Smoker: “[…] every prisoner got a letter saying […] as of November 2018, there’ll be a smoking ban coming […] there’s a poster […] it was like, nine months go to, eight months to go, seven months to go. So, they are giving you plenty warning, like, this is actually happening. So, there's nobody’s got an excuse now to say, ‘Oh I didn’t know about it’, like, everybody knows about it.”

Key findings from the interviews are presented below under two main themes: reactions to the impending smokefree prison policy, and opportunities, challenges and (participant) recommendations for policy implementation.

### Reactions to the impending smokefree prison policy

Opinions about the impending smokefree prison policy were on a continuum, with some participants occupying different positions on this continuum at different points in the interview. At one end of the continuum, participants expressed predominantly negative views on the basis that smokefree policy was perceived to be unfair in restricting the “freedom” to smoke and removing a substance associated with benefits (e.g. pleasure, relaxation, stress relief) as well as health harms:

WP4.D09. Smoker: “I think it's [smokefree prison policy] shocking, I think if you want to smoke it should be your ‘right’ to smoke […] a lot of people use [smoking] as a coping mechanism […] take that away from people I don't know what that's going to do.”

At the other end of the continuum, participants expressed more positive views about the smokefree policy, for personal reasons and, in some cases, because of the perceived potential benefits to the wider prison population (and for prison staff) – assuming smoking cessation support and treatments were available in prison. Some regarded the smokefree policy as an opportunity to finally succeed in quitting smoking:

WP4.F2.Smoker: “Probably, a lot of prisoners are similar to me and going ‘I’m glad that they’re going to do it, it’ll give me a chance to get off it [smoking]’, because I don’t think anybody really likes smoking, do you know what I mean? It’s enjoyable while you’re smoking but when you actually look at it and think about it I think everybody would go ‘I can’t believe I’m actually doing this!’ […] it’s so bad for you.”

Some participants expressed complex or nuanced views on the smokefree policy, simultaneously acknowledging and deliberating on perceived positive and negative aspects of prohibiting smoking in prison:

WP5.B39. Service User: It depends on what way you’re looking at it. For me personally I think, I think it is a good idea for people’s health. But at the same time I think people should be allowed to do what they want to do with respect to smoking, something that’s legal if you’re over 16.

As illustrated by the quotes below, participants across the continuum expressed concerns about potential adverse consequences of smokefree policy, including potential hardship or challenges for smokers (particularly new admissions, individuals on long-term sentences or those they considered vulnerable), possible violation of prison rules (e.g. sale of contraband tobacco) and perceived risks of conflict or violence:

WP4.B05.Service user: ‘[…] if you've been smoking for 30 years and you've got your routine, and routine is big things in prison, especially with lifers, […] they’ll get really upset when people mess up that routine.”

WP4.E04.Smoker: “[…] stopping smoking in prison…that won’t bother me. But you’ll find the rest of them will. And then that will affect me and that’s the part that worries me. The last thing I want is [other people] running about […] fighting and all the rest of it.”

WP4.D05.Ex-smoker: “[…] the prisoners will find another way of getting [tobacco]. That’s the nature of things. Then when it becomes scarce like that it becomes valuable. When it becomes valuable it creates prisoners getting into debt…”

At the time of the interviews, some people in custody were responding to the impending smokefree policy by proactively contemplating, planning or trying to make changes to their smoking behaviour. A key factor driving such changes was wanting to retain personal agency (“*I wanted to stop while it was still my choice rather than being forced into it*” WP5.E30.Ex-smoker), as well as to make it easier to manage without tobacco post implementation. This was particularly evident in the WP5 interviews which occurred some months before the smokefree policy came into force. Several participants said they would not have been trying to reduce or stop smoking if a smokefree policy was not imminent:

WP5.A11.Service user: “I think most folk are only really doing it [trying to stop smoking] as well just because of that ban, ken. Like, if it wasn’t for that, I don’t think anybody would really be giving it a second thought.”

Conversely, other people in custody were reportedly deploying avoidance strategies in light of the impending policy, at least at the time of these interviews. These included: believing or hoping that smokefree rules would not be actually implemented as planned; deciding to “deal” with the smokefree policy only “when it happens”; or speaking about some people’s intentions to continue smoking illicitly (although there were also concerns this may lead to sanctions and increased debt).

While it was implied that implementation of the smokefree policy would (mostly) necessitate smoking abstinence in prison, some participants raised factors which may influence return to smoking after release from (smokefree) prison, including the strength of a person’s desire to not smoke; the length of the period of smoking abstinence/cessation in prison; and experience of cravings:

WP5. C20. Smoker: The short termers [and] guys on remand. They’ll be saying…’I’ve only got a few months and then I’ll be out there for good’, so I’d imagine […] a lot of them will be smoking again not too long after [leaving] prison.”

With respect to social and situational factors, being around non-smokers and participation in work/other activities were perceived to support non-smoking out of prison, while the ready availability of tobacco, drugs and alcohol in wider society was mentioned as a potential driver back to smoking:

WP4.A03. Service user: “I'll do it [abstain from smoking] while I'm in here, but I […] it’s when I get out, you know, sitting in company and that, having a drink or whatever […]”

### Opportunities, challenges and participant recommendations for policy implementation

Participants described several issues which might present opportunities or create challenges for implementing smokefree prison policy. These are presented below under three headings: role of smoking in prison, prison smoking cessation services and motivations for quitting smoking for people in custody. The section concludes by providing an overview of participants’ suggestions for aiding policy implementation.

#### Role of smoking in prison.

The role of smoking in prison has the potential to make implementation of smokefree prison policy particularly challenging. As seen elsewhere, the use of tobacco in prisons in Scotland was often habitual and an important part of participants’ daily lives. For example, some described established smoking routines or rituals in terms of their first cigarette of the day, smoking after meals and when socialising with other people in custody. It was explained that breaking ingrained smoking habits could be especially challenging inside prison, even among those who had already made an active quit attempt. This was attributed to a perceived lack of replacement activities, the normative nature of being a smoker in this context and the sense of comfort it provided some participants to keep to a fixed routine:

WP5.D2. Service user: “And that’s six days on the Champix, and I don't feel any desire for a cigarette, I've no craving there. If it [tobacco] was there, I'd probably roll one without even thinking about it, just through the habit, kind of thing. So my problem now is, it won't be so much not smoking a cigarette, it'll be breaking the habit of, the routine you’ve had for years, kind of thing. You get up in the morning, you make your tea, you sit at your table, you roll a cigarette, and you have a cigarette with your tea […]”

Boredom was frequently cited as a driver of smoking in prison. Participants used smoking to pass or break up time, particularly while locked in their room (cell) when it was perceived there was little to do beyond drinking tea or coffee, watching TV and smoking:

WP5.C21.Service user: “[…] it’s [smoking] a distraction, isn’t it? It’s just something […] you’re bored…sitting watching the telly […] I’ll have a wee cup of coffee and a wee smoke and it’s a wee […] it’s a pleasure.”

Alternative ways of keeping their hands and minds occupied, both in and out of their rooms, had helped some people in custody to reduce or quit smoking and included physical exercise, writing letters, puzzles or games and participation in work, programmes or activities:

WP4.F5.Smoker “[…] I've cut right down [since having a job in the prison gym], I was smoking like 20 cigarettes a day, 20 burns a day. And now I'm only smoking I think it's about 11, and I feel like I can run more in the gym, I can lift more weights, when I'm playing football I'm not out of breath as much […]”

Another potential driver of tobacco use in prison was the high levels of stress and anxiety which some participants experienced during their sentence as a result of factors such as appearing in court, arguments with other people in custody or staff, or receiving “bad” news. In the absence of alternative coping strategies, such as being able to go for a walk or spend time with friends and family, it was suggested that smoking could help with management of mood and emotions:

WP5.A8.Smoker: I stopped for eight weeks, and I stopped [again] for six weeks. But it's, when you get bad news, or something happens in the hall [residential wing], and you just can't hold that anger in. So rather than explode, you go and have a roll-up, and then that leads to another.

#### Prison smoking cessation services.

The overall work of prison smoking cessation services was widely praised by participants who had attended sessions and four key strengths of these services were described, which might support implementation of smokefree policy. First, for some participants, regular CO monitoring facilitated compliance and provided confirmation of the health benefits of abstinence:

WP5.E29.Ex-smoker “[…] the smoking [cessation] programme for me wasn't about so much the products, it was more along the lines of somebody just checking you [via CO monitoring].”

Second, expert advisors were seen as a valued source of information and support for smoking cessation:

WP5.B39.Service user: “[…] she’s [smoking cessation advisor] been really good […] She’s like, she’s gave me great tips, she’s sent me colouring in stuff and puzzles through the post, just the first sort of few weeks just to, she’s been very supportive, very, like didn’t let it get you down, she’s still trying with me although for the past four weeks I’ve been, because for about three or four weeks I didn’t smoke at all and then, no I had one roll up within that whole time […]

Third, some participants appreciated the opportunity in group-based sessions to meet others who were trying to give up and talk about ways of overcoming barriers to smoking cessation (in prison):

WP5.C26.Service user: “[…] for me, that’s been quite helpful, hearing the other guys that are successful, that are really trying. And have stopped the same as me. And how they’ve filled their time in or their head’s been at the time […].I found that quite helpful.”

Finally, some participants spoke about making greater progress in their quit attempts after services prescribed a form of stop-smoking pharmacotherapy which they found effective:

WP5.E31.Ex-smoker: “[…] the wee mint lozenges and the patches helped me, but other guys prefer just patches and inhaler, other guys just prefer the mints […] I think it’s down to individual preference.”

At the same time, four main challenges in accessing and deriving maximum benefit from prison smoking cessation services were identified. First, some in the WP4 interviews expressed beliefs that conventional smoking cessation support or treatments did not work for them personally or that willpower alone was key to successful cessation. It is possible that such views could inhibit meaningful engagement with services by some smokers who might otherwise benefit.

Second, some participants wishing to use the specialist cessation service reported unpredictable or lengthy waiting times, which had hindered quit attempts:

WP4.C06.Service user: “[…] if you’re saying to yourself I want to try and give up […] and then you put your name down thinking it’s going to be a week, two weeks, three weeks down the line and it’s two […] going in to two months you’d end up saying […]’Give us a fag! Forget it!’”

Third, some participants indicated that aspects of the behavioural support provided by the stop smoking service had not met their needs, in terms of pace, format of sessions or the level of support given by advisors. CO monitoring could create worry for participants due to the risk that individuals might be asked to leave the cessation service sessions if readings exceeded the cut-off:

WP4.B03.Service user: “all they [cessation services] do […] is give you maybe a box of patches, mints, the inhaler thing or the spray. […] they give you the product and let you do it by yourself […]. There’s not enough support, like […] I don’t know, like, it’s a habit so you need […] it’s not […] it’s […] I wouldn’t say it is the same as a drug habit, but it’s still a habit you need help.”

Finally, there were reports of delays and mix-ups regarding the prescribing of medications to support smoking cessation in prison. Variation in the use of these medications across services could create difficulties if people were transferred between prisons:

WP4.A3.Service user: “They’re slow in here with the prescriptions. Uhm-hmm. They want you to stop smoking, right, so you do try. And then they don’t come across with tablets. They’re being late with giving you your next prescription. And that can knock you, kind of, back.”

The data reflected the considerable efforts that were already underway in some prisons by the time of the WP5 interviews (in May–June 2018) to enhance capacity and improve delivery of smoking cessation services and the distribution of NRT, to assist in the lead up to smokefree rules:

WP5.E32Ex-smoker: I put an application in [to access smoking cessation service]. I was lucky that they’d started doubling the numbers at that point, so I got on it relatively easy.

#### Motivations for quitting smoking in people in custody.

As noted above, participants expressed different levels of motivations for wanting to quit smoking or not, presenting both opportunities and challenges for smokefree policy implementation. Several health-related factors were important drivers for smoking cessation in people in custody, including a desire to improve health, experience of smoking-related health problems, or illness or death of a family member who smoked:

WP4.C01.Smoker: “ […] I find it hard to breathe. That’s why I want to stop […] And that’s the only reason. Like, smoking doesn’t bother me. I’ve done it all my days […] I don’t like it, but it’s what I do, isn’t it. But I can’t breathe. So I need to stop.”

Some participants said that making positive changes in other behaviours, such as drug use or physical activity, could provide impetus to try to stop smoking:

WP4.B5.Service user: I think that [going to the prison gym] was more what prompted me to do it [try to quit smoking] as well, because it was like doing sprints in the hall, you know, you're struggling, you know, you're like, I can’t keep […] Or also the fact you're seeing it as a bit pointless because you're not really going to progress if you're still, if you're plating quite a lot of food and then if you're still smoking as well…

Financial considerations were also potentially important in facilitating attempts to reduce or stop smoking in prison:

WP5.D6. Service user: “A complete honest reason [for trying to quit], I couldn't afford it [tobacco] […].okay and obviously my health as well but it was I couldn’t afford to smoke. You know, I’m on a £10 [weekly] wage. I like my coffee, I want to buy the odd treat, I want to buy shampoo, I want to buy normal things. So, when I was smoking that took up the whole budget, so it was either smoke or get other bits of fruits and things.”

A final factor potentially incentivising quitting for people in custody was positive social influence from peers or family members. For example, some participants said they wanted to quit partly for their partners or children or because others in their family had managed to give up:

Interviewer: What made you decide, right, I'm gonna give it a go, I'm gonna try and stop again?

WP5.A15.Smoker:“To stop? Because I've got two young kids, and I thought, well for me, personally for my health, and I don't want them to see me smoking, when I get out, know what I mean? Just to stop for health issues, and all. Because none of my family really smoke, even my partner, he doesn’t smoke either, so it's really me.”

By contrast, factors potentially influencing low levels of motivations to quit smoking among some people in custody were related to associations of smoking with physical and psychological rewards (e.g. relief of cravings), pleasure and mood management. There were also some misunderstandings of the benefits of quitting or the health risks of smoking. For example, one older smoker suggested that quitting smoking was futile since the ‘*damage is done’* (WP4.A5.Smoker). Another participant spoke about a time in the past when he believed that he was not at risk of cancer because his older relatives who smoked had not developed cancer.

#### Participant suggestions for aiding policy implementation.

When participants were asked how they thought that implementation of smokefree policy could be aided in prison (given that a decision had already been taken to prohibit the use of tobacco in Scottish prisons from 30 November 2018), support was expressed for measures such as improving access to the prison smoking cessation service; resolving issues in respect of prescribing of medications to support cessation; introducing e-cigarettes; providing more activities to replace smoking or keep someone occupied; and expanding communications and dialogue with people in custody about the approaching policy change. People in custody also expressed a concern that suitable strategies should be in place post-implementation to help smokers, including opportunities to access nicotine substitutes and behavioural support for new arrivals into prison:

WP5.C22. Ex-smoker: “I think they would need some nicotine replacement straight away as they come in. Like not leaving it […] Because most people that come in are usually alcoholic, drug dependent so if they’re already coming off them, adding nicotine into the mix as well which […]”

WP5.D1.Service user: I think […] they should […] reconsider […] what kind of product you buy to help you stop smoking […] they have to get a decent vaporiser [e-cigarette]

## Discussion

This study exploring perspectives of smokefree prison policy in people in custody in Scotland contributes to the evidence base available to help inform jurisdictions that are considering removing tobacco from their prisons in the future. The overall study of which this is a part has several novel features, including that data are being collected across a whole prison system before, during and after transitioning to becoming smokefree on 30 November 2018. The data collected for this analysis gives voice to a diverse range of people in custody (with respect to sex, remand or convicted status, sentence length and use of prison smoking cessation support) and prisons (in terms of their size, population mix, geography and security level) in the months leading up to policy implementation. Findings from the study have helped to inform the process of creating a smokefree prison estate in Scotland.

Prior to the announcement of the decision to implement smokefree prison policy in Scotland (in Phase 1), levels of support for smokefree policies were lower among people in custody than staff (Brown *et al.*, 2018). This subsequent (Phase 2) study found that opposition to prison smokefree policy among people in custody centred on concerns about the fairness and legitimacy of smokefree rules and apprehension about the potential adverse impact on smokers. This is consistent with previous qualitative studies exploring prison smokefree policy from the perspective of people in custody (Dugdale *et al.*, 2019; Woodall and Tattersfield, 2017). As we have discussed elsewhere (Brown *et al.*, 2018), it is important that potential concerns among stakeholders are mitigated in preparation for smokefree prison policy as far as possible. A novel finding of this study is that, at least in this pre-implementation period, several participants expressed more positive or nuanced views on prison smokefree policy due to anticipating potential benefits of smokefree rules, for themselves and prison and staff populations. One potential reason for differences between the findings of this study and previous research might be differences in the ways in which the smokefree prison policy was announced and implemented in Scotland, in comparison with other countries including England and Wales. Scotland took the bold decision to prohibit the use of tobacco in all 15 prisons simultaneously (from midnight on 30 November 2018). In the period leading up to prisons becoming smokefree in Scotland, national and local communication campaigns (including count down posters in residential halls and other parts of all prisons, and use of prison radio/print media) were undertaken to raise awareness in people in custody, staff and visitors of the date for the forthcoming national legislative change and to signpost smokers to enhanced smoking cessation services. Evidence from across TIPs suggest that communications were very effective in raising awareness, even if people in custody did not always fully agree with the decision to implement comprehensive smokefree rules, and at times wanted more detail. By contrast, Dugdale *et al.*’s (2019) study, conducted in four prisons in the north of England, found that ‘awareness about the ban and the implications of this appeared generally poor’ among participants (Dugdale *et al.*, 2019, p. 123). Differences in the size of the prison estate in England and Scotland may have been a key factor influencing variations in the ways in which smokefree policies were implemented across the UK, although that is not to diminish the implementation success achieved by the Scottish Prison Service (The Scotsman, 2019) and key partners including the NHS. In addition, this study might have been able to capture a more diverse range of perspectives on the forthcoming smokefree policy by using one-to-one interviews rather than focus groups, as it appeared that some participants were more comfortable challenging some of the prevailing narratives about smokefree rules within the privacy of an interview.

The findings, including participant recommendations, highlight several factors that might support successful implementation of smokefree prison policy in other jurisdictions in the future. The implications of our study are consistent with other literature on smoking cessation in prison (Djachenko *et al.*, 2015; Eadie *et al.*, 2012; Richmond *et al.*, 2009) and implementation of the smokefree prisons (Foley *et al.*, 2010; Hefler *et al.*, 2016; Collinson *et al.*, 2012). First, the importance of ensuring that imprisoned smokers have access to low cost or free evidence-based smoking cessation interventions, is widely recognised (Butler and Yap, 2015; Donahue, 2009; Ritter, 2014) and is confirmed by this study. Given that imprisoned smokers might feel some ambivalence or even hostility about *enforced* smoking abstinence, it is important that considerable efforts are made to reduce barriers to engagement with services – as far as resources permit. This includes delivering communication campaigns in prisons that are clear, wide-reaching and connect with people in custody’s expressed values and aspirations in relation to health and well-being, personal finances and family life. Using multiple channels and involving frontline staff and peer mentors in this process, as happened in Scotland, has the potential to increase the effectiveness of communication. Consideration could be given in the future to developing family-based smoking cessation interventions in prisons, aligning with wider aspirations to strengthen connections and quality of relationships between people in custody and their families (Scottish Prison Service, 2017b), as well as reaching out into the community to reduce inequalities in smoking and health. Second, the findings support the need for campaigns to acknowledge and sensitively challenge strong associations of smoking with pleasure and stress relief in the prison setting. At the same time, the role of structural factors in shaping health behaviours is highlighted in participant accounts of smoking, and these need to be considered when introducing measures to facilitate smoking abstinence. As a minimum, it is important to ask people in custody for their ideas for low-cost measures that would make a difference to them in managing without tobacco, such as more games/activities/hobbies that can be used to pass and break up time spent in their rooms (cells) or offering greater access to valued facilities such as the prison gym. Third, our findings suggest the need for measures to minimise the numbers of people returning to smoking after leaving a smokefree prison, e.g. establishing systems for onward referral of prison populations to community smoking cessation services.

A key strength of this study is that interviews were conducted with a relatively large and diverse sample of people in custody at an important moment when prisons where preparing for a cross-national implementation of smokefree prison rules, at a time of high rates of smoking (Scottish Prison Service, 2015) and entrenched smoking norms. This paper has therefore been able to describe in detail some key opportunities and challenges of tackling smoking in the prison setting, providing evidence that may be valuable for jurisdictions seeking to implement similar smokefree policies.

An important limitation of the study is that there may be differences in smoking-related opinions and experiences of people in custody who opted to take part in interviews compared with those who did not. Another limitation is differences in the intended aims and data collection and analysis processes for WP4 and WP5 (as described in “Methods”). However, our preliminary, separate, analyses of the two data sets identified very similar themes, and we believe that the depth of insight generated by combining the two datasets outweighs any minor methodological differences between the two work packages.

In conclusion, our study exploring smokefree prisons from the perspectives of people in custody has several novel features which extend the evidence base. The findings suggest several factors that might support successful implementation of smokefree prisons in other jurisdictions. They include (increased provision of and access to) evidence-based smoking cessation support in advance of smokefree policy, effective and comprehensive communication campaigns, consideration of broader structural determinants of health in prison and ongoing measures to reduce rates of return to smoking post release.

## Acknowledgment

We are grateful to the people in custody who took part in the interviews, staff within the prison service who participated in focus groups and SPS staff who assisted with the study and facilitated access. We gratefully acknowledge the contribution of our co-investigators in TIPs grant to the overall design of the study (Helen Sweeting, Sean Semple, Linda Bauld, Jill Pell, Alastair Leyland, Peter Craig, Kathleen Boyd, Evangelia Demou, Philip Conaglen and Emily Tweed. We are particularly grateful to Linda Bauld for her contribution to WP5 of TIPs.

*Funding*: The Tobacco in Prisons study is funded by the National Institute for Health Research (NIHR) [Public Health Research Programme, project number 15/55/44]. The views expressed are those of the author(s) and not necessarily those of the NIHR or the Department of Health and Social Care.

## Figures and Tables

**Table 1:** Sample characteristics

Characteristic	Categories	WP4	WP5	Combined total
Sex	Female	4	12	16
	Male	29	32	61
Remanded/convicted status	Convicted	30	35	65
	Remanded	3	2	5
	Missing data	0	7	7
Sentence length	Short-term	14	18	32
	Long-term	16	16	32
	Not applicable	3	2	5
	Missing data	0	8	8
